# Adaptive Waveform Design for Cognitive Radar in Multiple Targets Situation

**DOI:** 10.3390/e20020114

**Published:** 2018-02-09

**Authors:** Xiaowen Zhang, Xingzhao Liu

**Affiliations:** School of Electronic Information and Electrical Engineering, Shanghai Jiao Tong University, Shanghai 200210, China

**Keywords:** cognitive radar, waveform optimization, target detection, target estimation, multiple targets

## Abstract

In this paper, the problem of cognitive radar (CR) waveform optimization design for target detection and estimation in multiple extended targets situations is investigated. This problem is analyzed in signal-dependent interference, as well as additive channel noise for extended targets with unknown target impulse response (TIR). To address this problem, an improved algorithm is employed for target detection by maximizing the detection probability of the received echo on the promise of ensuring the TIR estimation precision. In this algorithm, an additional weight vector is introduced to achieve a trade-off among different targets. Both the estimate of TIR and transmit waveform can be updated at each step based on the previous step. Under the same constraint on waveform energy and bandwidth, the information theoretical approach is also considered. In addition, the relationship between the waveforms that are designed based on the two criteria is discussed. Unlike most existing works that only consider single target with temporally correlated characteristics, waveform design for multiple extended targets is considered in this method. Simulation results demonstrate that compared with linear frequency modulated (LFM) signal, waveforms designed based on maximum detection probability and maximum mutual information (MI) criteria can make radar echoes contain more multiple-target information and improve radar performance as a result.

## 1. Introduction

Cognitive radar is a recently proposed system concept, one of whose most important characteristics is its closed-loop operation [1]. The feedback structure from the receiver to the transmitter enables the transmit waveform optimized based on all available knowledge about the target and environment online, to improve its performance of detection, estimation, and identification [2,3]. Conventional radars feature pre-programmed waveforms and receive processing, purposefully selected for specific types of targets in predefined environments. In complex propagation and interference environments, conventional radar may not fully exploit the flexibility of the whole system so as to provide satisfactory detection, tracking, and recognition performance. In contrast, CR is able to adjust its work mode, transmit waveform, and signal-processing approach, which will make it perform well in the face of different challenges in a complicated environment. It is generally known that the Cognitive Radar Technology (CRT) is correlated with Electronic Warfare (EW), Electronic Support Measures/Electronic Intelligence (ESM/ELINT) class, especially in military applications. The identification and classification processes play a very important role in radar task [4,5], which are strongly correlated with waveform optimization design.

Waveform optimization design is an emerging topic in signal processing with applications in many areas, such as radar, sonar, communications (such as covert communications and spectral co-existence) [6,7], and so on. In past several years, knowledge-based (KB) transmit waveform design has received intensive attention [8]. Traditionally, optimal waveform design is determined by target and interference models, as well as the design criterion. The target models can be divided into point targets and extended targets. The classical radar target model assumes a far-field point source target. When the radar transmits a relatively narrow bandwidth signal, the range span of the target is well within a single range cell. Thus, a point target model is often valid. But, as the waveform bandwidth *B* becomes comparable to c/2Δz, where *c* is the speed of light and Δz is the spatial extent of the radar target in range, the point-target model does not accurately present the behaviour of radar scatters. In fact, the return must be viewed from several points in an extended region of space and the received signal is the sum of multiple delayed versions of the transmitted waveform. Targets exhibiting such scattering behaviour are called extended targets [9]. The interference might comprise clutter, noise, or both of them. Clutter is assumed to be signal-dependent, such as unwanted ground returns and environment clutter, whereas noise is signal-independent. Current design criteria include the maximum signal to interference plus noise ratio (SINR) criterion, the maximum detection probability criterion, the minimum mean square error (MMSE) criterion, the maximum MI criterion, and so on. The selection of these criteria usually depends on the task of the radar system. For example, for the task of detecting a particular target, the output signal to noise ratio (SNR) should be maximized, the maximum SINR criterion or the maximum detection probability criterion is often selected. Radar detection has attracted enormous interest in the last decades. Many excellent works dealing with the design and performance analysis of suitable detectors under several specific settings were appeared in adaptive detection [10,11,12]. As it is generally known, the detection performance is highly relevant to waveform optimization design. Earlier work in waveform design for target detection includes the work in [13,14,15,16,17,18,19,20,21,22,23]. The SINR of echo waveform can be maximized by joint optimizing the waveform and receiver impulse response, which can further improve the target detection performance [13]. A novel iterative algorithm is proposed by the authors [15] to joint optimization of waveforms and receiving filters in the multiple-input multiple-output (MIMO) radar such that the detection performance can be maximized. For polarimetric radar, an iterative optimization procedure is developed by considering the worst case SINR at the output as the figure of merit to optimize the transmit signal under both a similarity and an energy constraint [16]. In order to maximize either the probability of detection of a given target class or the probability of correct identification between two target classes, the optimization of the full-polarization transmission waveform and the receiver impulse response is investigated in [17]. The work of Kay [19] presented optimal signal design for detection of Gaussian point target in Gaussian clutter. In addition, signal design for clutter rejection was considered in [23]. Considering the problem of target estimation, the estimation theoretic criterion or information theoretic criterion is usually utilized. The MMSE criterion intends to design the optimal waveform that minimizes estimation errors at the output of the MSE estimator [24]. Information theory has been applied in radar signal processing for over a half a century now. Bell proposed the method of using mutual information between target and radar echo to design transmit waveform, which can extract more target-information from the received measurements [9]. Henceforth, information theoretic criteria, especially mutual information and relative entropy (also known as Kullback-Leibler divergence), have been at the core of adaptive radar waveform design algorithms. In the radar system with multiple targets, Leshem used an information theoretic approach for estimating and tracking parameters of multiple targets under a noise background [25]. In [26], Goodman proposed the integration of waveform design with a sequential-hypothesis testing framework when hard decisions may be made with adequate confidence. Additionally, with the recent emergence of CR, which learns from its experience in addition to sensing and adapting, the concept of information preservation has become even more relevant in the radar receiver processing chain.

In CR, target detection and estimation employ feedback from the environment to tune the radar’s transmit waveform or the radar’s receive processing in such a way as to improve the performance in complex radar environments. The authors presented the initial cognitive framework for optimal waveform design in [27]. Based on the idea of cognitive tracking radar proposed by Haykin, an iteration method based on Kalman filtering (KF) is first proposed in [28] to estimate the TIR of extended target. Since then, research in adapting the cognitive waveform to unknown target and environment has flourished. To improve the reliability of detection decision, the author proposed a two-step detection signal process which is called estimation before detection in [29]. An intelligent algorithm, that is, genetic algorithm, was attempted to solve waveform optimization problem. The detection performance was improved by the precise estimating of TIR. However, little research has been done on CR waveform design for multiple targets in the presence of signal-dependent interference, i.e., clutter. In a multiple-target environment, especially in dense target environments, radar usually does beam combined processing in order to detect and track more targets. The waveform design is different from the single target situation. Moreover, when the clutter is stronger than the signal reflected from the target of interest, the target will be masked. Thus, research on target detection for CR in the signal-dependent interference is necessary.

In this paper, waveform optimization design for target detection and estimation in a multiple-target situation is investigated. First, a closed-loop signal processing model of CR with multiple extended targets, signal-dependent interference, as well as additive channel noise is modeled in the Fourier domain. Second, unlike most existing CR waveform optimization designs with constant prior information, unknown TIR is considered in this method. A Kalman filter (KF) is used to track the extended targets for estimating the TIR. TIR achieved by Bayesian estimation is used as the initial state of the Kalman filter. The estimation accuracy will be improved when the number of KF iterations increases. Third, the whole optimization process in a multiple-target environment is established to design the transmit waveform by maximizing the detection probability of the received echo at each KF iteration. The algorithm is proposed on the premise of ensuring the TIR estimation precision. A weight vector is introduced to control the importance of different targets. Both the estimate of TIR and transmit waveform can be updated at each step based on the previous step. Under the same constraint on waveform energy and bandwidth, the information theoretical approach is also considered. In addition, the relationship between the waveforms that are designed based on the two criteria is discussed.

This paper is organized as follows: Section 2 describes the signal processing model of CR system in a multiple-target situation. In Section 3, we formulate an iterative algorithm for target detection by maximizing the detection probability of the received echo on the premise of the TIR estimation precision. The information theoretical approach is also investigated under the same constraint on waveform energy and bandwidth. The relationship between the waveforms that are designed based on the two criteria is discussed. In Section 4, numerical simulations are conducted to present the energy distribution and performance of the proposed detection probability-based waveform, MI-based waveform and traditional LFM waveform. Finally conclusions are drawn in Section 5.

Notations used in this paper are defined as follows: symbols for vectors (lower case) and matrices (upper case) are in bold face. ${\left(\cdot \right)}^{H}$, ${\left(\cdot \right)}^{T}$, ${\left(\cdot \right)}^{*}$, diag{⋅}, Tr{⋅}, I, CN(0,R), ${\left\|\;\cdot \;\right\|}_{2}$, F, ∗ denote the complex conjugate transpose, the transpose operation, the complex conjugate operation, the diagonal matrix, the trace of a matrix, the identity matrix, the complex Gaussian distribution with zero mean and covariance being R, the $l_{2}$ norm, the Fourier transform, convolution, respectively.

## 2. System Model

**Figure 1:**
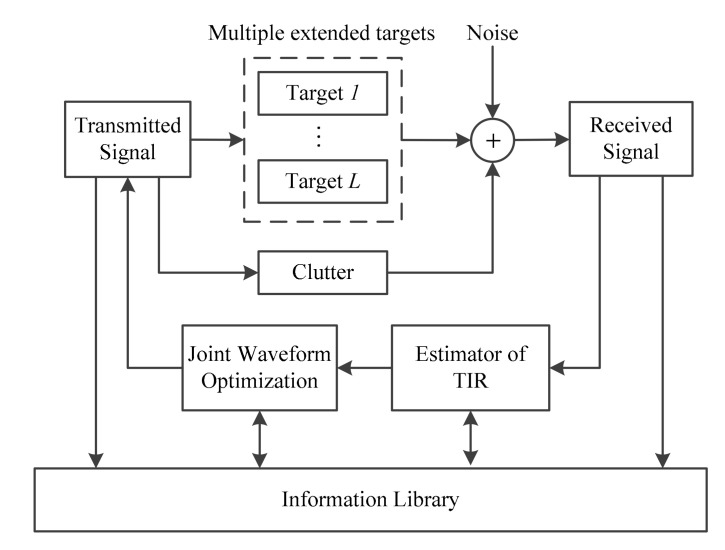
Signal processing model of CR system in a multiple-target situation.

The signal processing model of CR system in multiple extended targets situation is shown in Figure 1. The closed-loop cycle begins with the transmitter illuminating the environment. Radar returns produced by the environment are fed into the receiver. According to the TIR estimator and prior knowledge in the information library, the signal processor makes decisions on the optimal waveform. Meanwhile, information about the waveform and the environment will be updated in information library. In return, the transmitter illuminates the environment in light of the decision fed back by the receiver.

In the *k*-th cycle of CR, the transmit waveform is denoted by $s_{k}$, where $s_{k}={\left[s_{k}\left(0\right)\;\ldots \;s_{k}\left(M-1\right)\right]}^{T}$. The time interval of transmit waveform is $T_{s}=MT_{1}$, where $T_{1}$ denotes the sampling interval. We assume $s_{k}$ is energy-limited. Thus, the transmit energy constraint can be expressed as:

(1) $${\left\|s_{k}\right\|}_{2}{}^{2}=E_{s}$$

where *E_s_* is the energy of transmitted waveform. Let $g_{i,k}$ represent the TIR of the *i*-th target in the *k*-th cycle. According to [28,30,31], the extended target behaves as a linear time-invariant filter with random complex impulse response, where the complex amplitude of every cell is a zero-mean complex Gaussian variable. When the target backscattering surface is assumed to be rough compared with the wavelength of the radar carrier, the variables of different range cells are unrelated to each other. We assume $g_{i,k}$ is a zero-mean Gaussian random vector with full rank covariance matrix $\Sigma_{i,g}$. The extended targets are described by the wide sense stationary uncorrelated scattering (WSSUS) model [28], and the TIR of the *i*-th target is:

(2) $$g_{i,k}=e^{-T/\tau_{i}}g_{i,k-1}+u_{i,k-1}$$

where *T* denotes the pulse repetition interval (PRI) of the radar. $\tau_{i}$ denotes the temporal decay constant. $u_{i,k-1}$ denotes the excitation noise vector. $g_{i,k}$ and $u_{i,k-1}$ are independent. Assuming that the TIR is a second-order stationary, $u_{i,k-1}$ should be a zero-mean complex Gaussian vector with covariance matrix $\sum_{i,u}=\left(1-e^{-2T/\tau_{i}}\right)\sum_{i,g}$. The target scattering coefficient (TSC), which is the Fourier transform of TIR, can be expressed as:

(3) $$G_{i,k}=e^{-T/\tau_{i}}G_{i,k-1}+V_{i,k-1}$$

where $G_{i,k}=F\;g_{i,k}\in {\mathbb{C}}^{M\times 1}$ follows the complex Gaussian distribution $CN\left(0,\;R_{i,G}=F\;\sum_{i,g}\;F^{H}\right)$. $V_{i,k-1}≜F\;u_{i,k-1}\;\in {\mathbb{C}}^{M\times 1}$ denotes the Fourier transform of $u_{i,k-1}$, which follows the complex Gaussian distribution $CN\left(0,\;\left(1-e^{-2T/\tau_{i}}\right)R_{i,G}\right)$. The clutter impulse response (CIR) $c_{k}$ is a complex-valued, zero-mean Gaussian random vector [14,29] with full rank covariance matrix $\Sigma_{c}$. Let $n_{k}$ denote a zero-mean white complex Gaussian noise vector with covariance matrix $Q=\sigma_{n}{}^{2}I$. The components of noise are assumed to be independently and identically distributed (i.i.d). The received signal $y_{i,k}$ of the *i*-th target in the *k*-th cycle can be obtained as:

(4) $$y_{i,k}=s_{k}*g_{i,k}+s_{k}*c_{k}+n_{k}$$

It can be written in frequency domain as:

(5) $$Y_{i,k}=S_{k}G_{i,k}+W_{k}$$

where the M×1 vector $W_{k}=S_{k}C_{k}+N_{k}$, the diagonal matrix $S_{k}=diag\left\{{\bar{S}}_{k}\right\}\in {\mathbb{C}}^{M\times M}$, and ${\bar{S}}_{k}=F\;s_{k}\in {\mathbb{C}}^{M\times 1}$. Moreover, $C_{k}=F\;c_{k}\in {\mathbb{C}}^{M\times 1}$ follows the complex Gaussian distribution $CN\left(0,\;R_{C}=F\;\sum_{c}\;F^{H}\right)$, $N_{k}=F\;n_{k}\in {\mathbb{C}}^{M\times 1}$ follows the complex Gaussian distribution $CN\left(0,\;R_{N}=F\;Q\;F^{H}\right)$, and $W_{k}\in {\mathbb{C}}^{M\times 1}$ follows the complex Gaussian distribution $CN\left(0,\;R_{W,k}=S_{k}R_{C}S_{k}{}^{H}+R_{N}\right)$.

## 3. Optimization of Waveform Design

The transmit waveform can be adapted in response to the information regarding the radar environment, which is a key component of the CR system. In order to achieve higher estimation and detection performance, CR should transmit the waveform matched with the environment to illuminate the target. In this section, a Kalman filter is used to track the extended targets for estimating the TIR. Then, an improved algorithm is employed for target detection by maximizing the detection probability of the received echo on the promise of ensuring the TIR estimation precision. In this algorithm, a weight vector is adopted to control the importance of different targets. Under the same constraint on waveform energy and bandwidth, the information theoretical approach is also investigated. In addition, the relationship between the waveforms that are designed based on the two criteria is discussed.

### 3.1. Target Impulse Response Tracking

Since the TIR is unknown, we consider the problem of TIR estimation and prediction using Kalman filter. Bayesian estimation of the TIR is investigated as the initial state of Kalman filter. Conditioned on $S_{0}$, $G_{i,0}$ and $Y_{i,0}$ are jointly Gaussian distributed as:

(6) $$\left[\begin{array}{c} G_{i,0}\\ Y_{i,0} \end{array}\right]↔CN\left(\left[\begin{array}{c} 0\\ 0 \end{array}\right],\;\left[\begin{array}{cc} R_{i,G} & R_{i,G}S_{0}{}^{H}\\ S_{0}R_{i,G}{}^{H} & S_{0}R_{i,G}S_{0}{}^{H}+R_{W,0} \end{array}\right]\right)$$

Let ${\hat{G}}_{i,0|0}$ denotes the Bayesian estimate of $G_{i,0}$. When the cost function is defined as ${\left\|{\hat{G}}_{i,0|0}-G_{i,0}\right\|}^{2}$, the Bayesian estimate ${\hat{G}}_{i,0|0}$ will be a linear MMSE estimator which is given by the conditional mean of $G_{i,0}$ given $Y_{i,0}$ [32], i.e.,

(7) $${\hat{G}}_{i,0|0}={\left(S_{0}{}^{H}S_{0}+R_{W,0}R_{i,G}{}^{-1}\right)}^{-1}S_{0}{}^{H}Y_{i,0}$$

The Bayesian matrix is:

(8) $$\begin{array}{l} P_{i,0|0}=E\left\{\left(G_{i,0}-{\hat{G}}_{i,0|0}\right){\left(G_{i,0}-{\hat{G}}_{i,0|0}\right)}^{H}\right\}\\ \;\;=R_{i,G}-R_{i,G}S_{0}{}^{H}{\left(S_{0}R_{i,G}S_{0}{}^{H}+R_{W,0}\right)}^{-1}S_{0}R_{i,G} \end{array}$$

The MMSE of the i-th target for a given $S_{0}$ will be:

(9) $$MMSE_{i}=E\left\{{\left\|{\hat{G}}_{i,0|0}-G_{i,0}\right\|}^{2}\right\}=tr\left\{P_{i,0|0}\right\}$$

The recursion of Kalman filter are:

(10) $${\hat{G}}_{i,k|k-1}=e^{-T/\tau_{i}}{\hat{G}}_{i,k-1|k-1}$$

(11) $$P_{i,k|k-1}=e^{-2T/\tau_{i}}P_{i,k-1|k-1}+\left(1-e^{-2T/\tau_{i}}\right)R_{i,G}$$

(12) $${\hat{G}}_{i,k|k}={\hat{G}}_{i,k|k-1}+K_{i,k}\left(Y_{i,k}-S_{k}{\hat{G}}_{i,k|k-1}\right)$$

(13) $$P_{i,k|k}=P_{i,k|k-1}-K_{i,k}S_{k}P_{i,k|k-1}$$

(14) $$K_{i,k}=P_{i,k|k-1}S_{k}{}^{H}{\left(R_{W,k}+S_{k}P_{i,k|k-1}S_{k}{}^{H}\right)}^{-1}$$

where ${\hat{G}}_{i,k|k}$ denotes the estimate of $G_{i,k}$ based on k−1 measurement data, $P_{i,k|k}$ the MMSE matrix and $K_{i,k}$ the Kalman gain matrix.

### 3.2. Maximum Detection Probability-Based Waveform Optimization Design

In this subsection, waveform optimization design for multiple extended targets is discussed. The whole optimization process in multiple-target environment is established to design the transmit waveform by maximizing the detection probability at each KF iteration. The estimate value of the *i*-th TSC in the *k*-th cycle is ${\hat{G}}_{i,k}$. According to the signal model in Figure 1, the equivalent detection problem is given by:

(15) $$\begin{array}{l} H_{0}:Y_{i,k}=S_{k}C_{k}+N_{k}\\ H_{1}:Y_{i,k}=S_{k}{\hat{G}}_{i,k}+S_{k}C_{k}+N_{k} \end{array}$$

Furthermore, since all these vectors are complex Gaussian random vectors and independent of each other, the detection problem of Equation (15) is equivalent to:

(16) $$\begin{array}{l} H_{0}:Y_{i,k}\sim CN(0,R_{W,k})\\ H_{1}:Y_{i,k}\sim CN(0,R_{S,i,k}+R_{W,k}) \end{array}$$

where $R_{S,i,k}$ is the covariance matrices of $S_{k}{\hat{G}}_{i,k}$. The probability density function (PDF) under either hypothesis is a complex multivariate Gaussian so that:

(17) $$\begin{array}{l} p(Y_{i,k};H_{0})=\frac{1}{\pi^{M+1}\left|\det(R_{W,k})\right|}\exp(-Y_{i,k}{}^{H}R_{W,k}{}^{-1}Y_{i,k})\\ p(Y_{i,k};H_{1})=\frac{1}{\pi^{M+1}\left|\det(R_{Y,i,k})\right|}\exp(-Y_{i,k}{}^{H}R_{Y,i,k}{}^{-1}Y_{i,k}) \end{array}$$

where $R_{Y,i,k}=R_{S,i,k}+R_{W,k}$. The log-likelihood ratio test can be written as:

(18) $$l\left(Y_{i,k}\right)=Y_{i,k}{}^{H}\left(R_{W,k}{}^{-1}-R_{Y,i,k}{}^{-1}\right)Y_{i,k}\begin{array}{c} \overset{H_{1}}{>}\\ \underset{H_{0}}{<} \end{array}\eta$$

Equation (18) is equivalent to:

(19) $$l\left(Y_{i,k}\right)\;=\frac{{\left|Y_{i,k}{}^{H}R_{W,k}{}^{-1}S_{k}{\hat{G}}_{i,k}\right|}^{2}}{1+{\left(S_{k}{\hat{G}}_{i,k}\right)}^{H}R_{W,k}{}^{-1}\left(S_{k}{\hat{G}}_{i,k}\right)}$$

Ignoring the constants, then we have the test statistic:

(20) $$T(Y_{i,k})={\left|Y_{i,k}{}^{H}R_{W,k}{}^{-1}S_{k}{\hat{G}}_{i,k}\right|}^{2}$$

We assume $\sigma^{2}$ denotes the variance under H. Considering $T(Y_{i,k})$ under $H_{0}$ we have:

(21) $$\sigma_{0}{{}_{i}}^{2}=E[Y_{i,k}{}^{H}R_{W,k}{}^{-1}S_{k}{\hat{G}}_{i,k}]={\left(S_{k}{\hat{G}}_{i,k}\right)}^{H}R_{W,k}{}^{-1}\left(S_{k}{\hat{G}}_{i,k}\right)$$

Similarly under $H_{1}$ we have:

(22) $$\sigma_{1}{{}_{i}}^{2}=E[Y_{i,k}{}^{H}R_{W,k}{}^{-1}S_{k}{\hat{G}}_{i,k}]=\sigma_{i,0}{}^{2}+{\left|{\left(S_{k}{\hat{G}}_{i,k}\right)}^{H}R_{W,k}{}^{-1}\left(S_{k}{\hat{G}}_{i,k}\right)\right|}^{2}$$

The detection probability can be expressed as:

(23) PDi=PFAσ0i2σ1i2=PFA11+Di,k

where $D_{i,k}=\frac{{\left(S_{k}{\hat{G}}_{i,k}\right)}^{H}\left(S_{k}{\hat{G}}_{i,k}\right)}{tr\left(R_{W,k}{}^{-1}\right)}$, $P_{FA}$ denotes the false alarm probability. As can be seen, $D_{i,k}$ is the SINR of the *i*-th target in the *k*-th cycle. For the same $D_{i,k}$, the detection probability $P_{D}{}_{i}$ increased significantly with the increasing of $P_{FA}$. In multiple-target environment, we introduce a weight vector to control the importance of different targets. The normalized detection probability is defined by:

(24) $${\bar{D}}_{k}=\sum_{i=1}^{L}\alpha_{i}D_{i,k}$$

where L denotes the number of extended targets. The target weight vector $\alpha ={\left[\alpha_{1},\;\ldots \;,\alpha_{L}\right]}^{T}$, $\alpha_{i}\geq 0$ and $\sum_{i=1}^{L}\alpha_{i}=1$. Accordingly, the optimization problem can be formulated as:

(25) $$\begin{array}{l} \underset{{\bar{S}}_{k}}{\max}\;\;{\bar{D}}_{k}\\ s.t.\;\;\;\;{\left\|{\bar{S}}_{k}\right\|}_{2}{}^{2}\leq E_{s} \end{array}$$

Since the cost function is concave, we can use the Lagrange duality to solve the constrained optimization. The solution is shown as below:

(26) $${\left|{\bar{S}}_{k}\left(m\right)\right|}^{2}=\max\left[\frac{\sqrt{\frac{\sum_{i=1}^{L}\alpha_{i}{\left|{\hat{G}}_{i,k}\left(m\right)\right|}^{2}R_{N}\left(m,m\right)}{\lambda_{k}}}-R_{N}\left(m,m\right)}{R_{C}\left(m,m\right)},\;0\right]$$

where the parameter $\lambda_{k}$ is found by solving:

(27) $$\sum_{m=0}^{M-1}{\left|{\bar{S}}_{k}\left(m\right)\right|}^{2}\leq E_{s}$$

Thus, synthesizes target tracking and detection, the estimate of TIR and transmit waveform can be updated iteratively in each cycle of CR. The choice of target weight vector depends on the specific situation of targets. For example, when the multiple targets differ slightly, the weight will be evenly distributed. Over all targets, an average detection probability will be obtained. When the multiple targets differ greatly, we will only focus on the most significant target. The corresponding weight vector $\alpha ={\left[1,\;\ldots \;,0\right]}^{T}$, which is the same as the single target situation. Moreover, the trade-off of different targets is determined by α, which can be adjusted according to the requirement of radar performance in each cycle of CR.

### 3.3. Maximum MI-Based Waveform Optimization Design

In this subsection, the whole optimization process in a multiple-target situation is established to design the transmit waveform by maximizing the MI at each KF iteration. The estimate value of the *i*-th TSC in the *k*-th cycle is ${\hat{G}}_{i,k}$. Based on the definition of MI [9], we know that the MI of the *i*-th target is:

(28) $$I_{i,k}\left(Y_{k}\;|X_{i,k}\right)=H\left(Y_{k}\right)-H\left(Y_{k}|X_{i,k}\right)$$

where $X_{i,k}=S_{k}{\hat{G}}_{i,k}$. Thus, it can be written as:

(29) $$I_{i,k}\left(Y_{k}\;|X_{i,k}\right)=\sum_{m=0}^{M-1}\ln\left(1+R_{SINR_{i,k}}\left(m\right)\right)$$

(30) $$R_{SINR_{i,k}}\left(m\right)=\frac{{\left|{\bar{S}}_{k}\left(m\right){\hat{G}}_{i,k}\left(m\right)\right|}^{2}}{{\left|{\bar{S}}_{k}\left(m\right)\right|}^{2}R_{C}\left(m,m\right)+R_{N}\left(m,m\right)}$$

where $R_{SINR_{i,k}}\left(m\right)$ denotes the SINR spectral density. In multiple-target environment, we introduce the weight vector to control the importance of different targets. The normalized SINR spectral density is defined by:

(31) $${\bar{R}}_{SINR_{k}}\left(m\right)=\sum_{i=1}^{L}\alpha_{i}R_{SINR_{i,k}}\left(m\right)$$

Thus, the MI can be written as:

(32) $$I_{k}=\sum_{m=0}^{M-1}\ln\left(1+{\bar{R}}_{SINR_{k}}\left(m\right)\right)$$

Accordingly, the optimization problem can be formulated as:

(33) $$\begin{array}{l} \underset{{\bar{S}}_{k}}{\max}\;\;I_{k}\\ s.t.\;\;\;\;{\left\|{\bar{S}}_{k}\right\|}_{2}{}^{2}\leq E_{s} \end{array}$$

Since the cost function is concave, we can use the Lagrange duality to solve the constrained optimization. The solution is shown as below:

(34) $${\left|{\bar{S}}_{k}(m)\right|}^{2}=\max\left[0,B_{k}\left(m\right)\left(A_{k}-U_{k}\left(m\right)\right)\right]$$

where:

(35) $$B_{k}\left(m\right)=\frac{\sum_{i=1}^{L}\alpha_{i}{\left|{\hat{G}}_{i,k}\left(m\right)\right|}^{2}}{2R_{C}\left(m,m\right)+\sum_{i=1}^{L}\alpha_{i}{\left|{\hat{G}}_{i,k}\left(m\right)\right|}^{2}}$$

(36) $$U_{k}\left(m\right)=\frac{R_{N}\left(m,m\right)}{\sum_{i=1}^{L}\alpha_{i}{\left|{\hat{G}}_{i,k}\left(m\right)\right|}^{2}}$$

The constant $A_{k}$ is determined by the energy constraint ${\left\|{\bar{S}}_{k}\right\|}_{2}{}^{2}\leq E_{s}$. The proposed waveform optimization design is presented in Algorithm 1.

**Algorithm 1**: The proposed waveform optimization design algorithm.S1: Transmit initial LFM signal $s_{0}$, where ${\bar{S}}_{0}=Fs_{0}$, $S_{0}=diag\left({\bar{S}}_{0}\right)$.S2: Capture the signal reflected from the target and interference. The received waveform vector is $Y_{i,0}=\left(S_{0}{\hat{G}}_{i,0|0}+S_{0}C_{0}+N_{0}\right)H_{0}$.S3: Estimate the initialize the estimated TSC and MSE matrix of the target as ${\hat{G}}_{i,0|0}$ and $P_{i,0|0}$ by the received waveform vector $Y_{0}$ and transmit waveform matrix $S_{i,0}$.S4: Set the iteration index k=1, and the maximal number of iteration as $K_{\max}$.
S5: while $k\leq K_{\max}$ do
S6: Predict the estimated TSC ${\hat{G}}_{i,k|k-1}$ according to ${\hat{G}}_{i,k-1|k-1}$ by (10).S7: Update the MSE matrix $P_{i,k|k-1}$ according to $P_{i,k-1|k-1}$ by (11).S8: Define ${\hat{G}}_{i,k}=diag\left({\hat{G}}_{i,k|k-1}\right)$, solve for ${\left|{\bar{S}}_{k}\left(m\right)\right|}^{2}$.S9: Obtain ${\hat{G}}_{i,k|k}$ and $P_{i,k|k}$ with $S_{k}$, $Y_{i,k}$, ${\hat{G}}_{i,k|k-1}$ and $P_{i,k|k-1}$ by Kalman filter.S10: Let k=k+1.S11: end while

**Figure 2:**
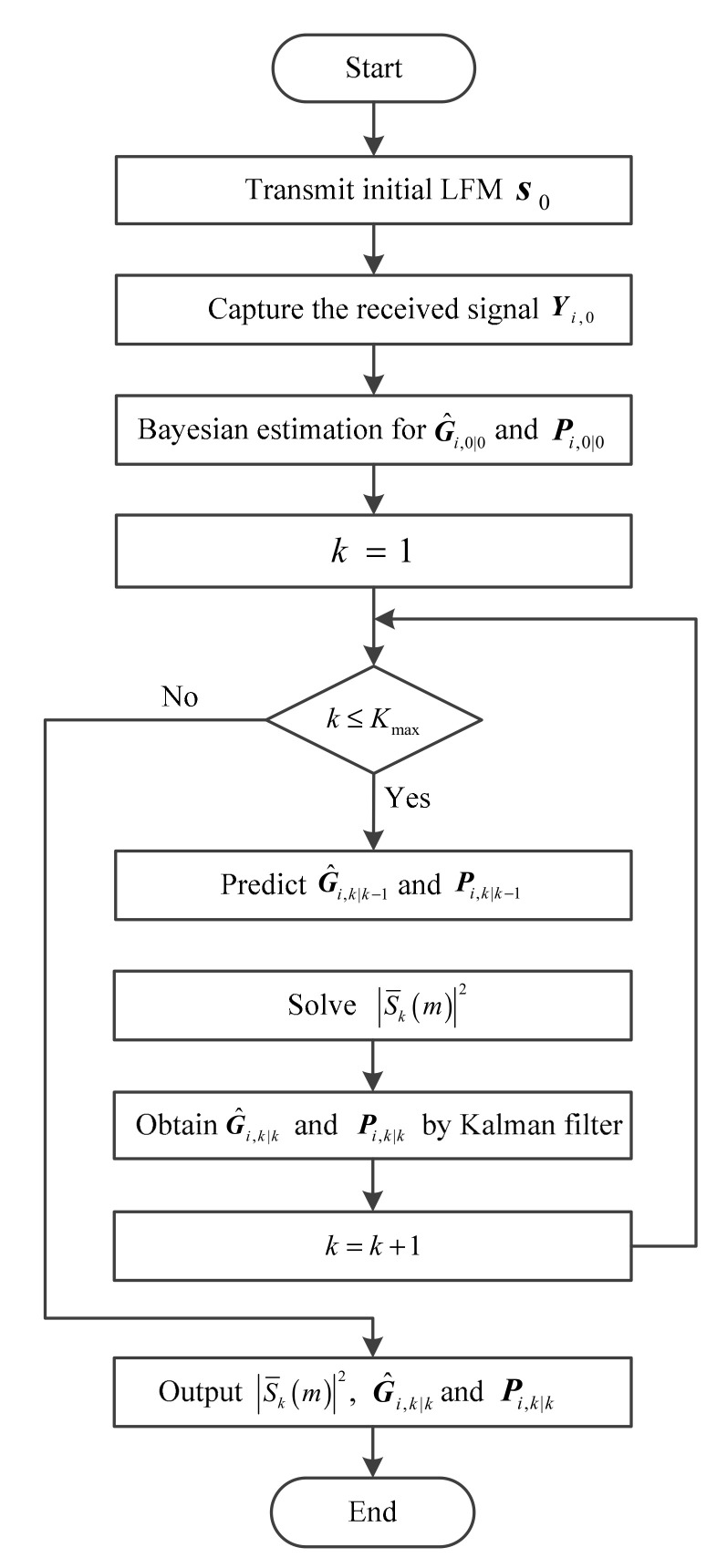
System flow chart of the proposed waveform optimization approach.

From the above derivation, the cost function designed based on MI criterion is a function of one designed based on the detection probability criterion. For frequency components with small coefficients in normalized SINR spectral density, the MI-based cost function is approximately equal to the detection probability-based cost function, however, for frequency components with large coefficients, the MI-based cost function is approximately the logarithm of the detection probability-based cost function. Above all, under the same bandwidth and energy constraints, the two criteria lead to the similar optimal waveform. The overall computational complexity of the proposed algorithm is linear with respect to the number of iteration. Due to the computation of the inverse matrices in Equation (14), the complexity of each iteration is in the order of $Ο\left(M^{3}\right)$ [33]. It should be noted that the knowledge of environment is unknown originally. Since the power spectrum of LFM waveform is distributed uniformly in the whole frequency band, we choose LFM waveform as the initial transmit waveform $s_{0}$. The proposed waveform optimization design algorithm is given above. The system flow chart of the proposed waveform optimization approach is shown in Figure 2.

## 4. Numerical Results

In this section, simulations are carried out to show the performance improvement of the proposed method. First, we show the power spectrum distribution of transmit waveforms designed based on the detection probability method and MI method, respectively. Then, the estimation, SINR, detection probability, and MI performance of CR are compared with those of traditional radar. Radar with adaptive transmitted waveforms is defined as CR, and radar with fixed LFM waveform is defined as traditional radar.

The simulation conditions are assumed as follows: two targets with different covariance matrixes of TSC are taken into consideration. The total bandwidth is 30 MHz. The time interval of transmit waveform is 0≤t≤10 μs. Here, we set the sampling frequency to 60 MHz. The PRI of transmit waveform is T=8 ms, the temporal decay constant is τ=0.8 s, the total transmit power is $E_{s}=1$ (energy unit), and the number of KF iterations is 30. Here, LFM signal is fixed waveform with constant envelope in every cycle. PD_Design1, PD_Design2, and PD_Design3 denote waveforms designed by the detection probability method. MI_Design1, MI_Design2, and MI_Design3 denote waveforms designed by the MI method. Parameters $\alpha_{1}$ and $\alpha_{2}$ of designed waveforms are shown in Table 1. The performance of LFM waveform is utilized for comparison.

**Figure 3:**
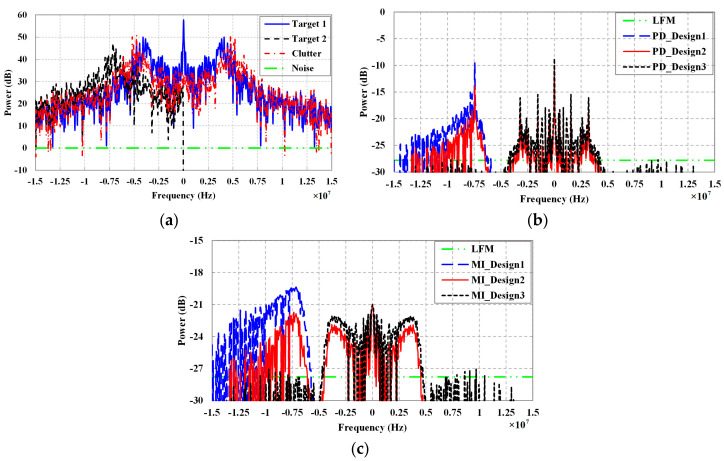
Power spectra: (**a**) Power spectra of targets, clutter, and noise; (**b**) Power spectra of detection probability-based waveforms; (**c**) Power spectra of MI-based waveforms.

Figure 3a shows the actual power spectra of targets, clutter, and noise, where the power spectra are defined as ${\left|G_{1,k}\right|}^{2}$, ${\left|G_{2,k}\right|}^{2}$, ${\left|C_{k}\right|}^{2}$, and ${\left|N_{k}\right|}^{2}$, respectively. The power spectrum distribution of transmit waveforms designed based on detection probability and MI criteria in the final KF iteration are depicted in Figure 3b,c, respectively, where the power is defined as ${\left|{\bar{S}}_{k}\right|}^{2}$. It can be found that the spectrum distribution of both PD_Design1 waveform and MI_Design1 waveform mainly concentrates in bands where the power of Target2 is large and the clutter power is small. Thus, the echo of waveform will contain more target information. Likewise, the spectrum distribution of PD_Design3 waveform and MI_Design3 waveform mainly concentrates in bands where the power of Target1 is large. The power spectrum of Design2 waveform is between Design1 waveform and Design3 waveform. As expected, the power spectrum of LFM waveform is constant. In addition, the impact of parameters $\alpha_{1}$ and $\alpha_{2}$ in the proposed method on waveform spectrum design can be obtained clearly. It is interesting to note that compared with LFM waveform, although the distributions of both detection probability-based and MI-based waveforms are more concentrated and significant, the energy allocation of the two waveforms is different in these intervals. Detection probability-based waveform tends to concentrate the main energy in a few narrow frequency bands. However, MI-based waveform tends to distribute its energy over some relatively wide bands. 

From the physical aspect, for waveform design in signal-dependent interference, the behavior of detection probability-based and MI-based optimal waveforms is modified to account for clutter. The clutter-compensating nature allows the waveforms to de-emphasize frequency components where clutter is strong and emphasize them where the target power is large. From the aspect of mathematics, this simulation validates well the relationship of detection probability criterion and MI criterion that are obtained in Section 3. Clearly the MI spectral density is always nonnegative due to the addition of one to the normalized SINR spectral density. For frequency components with small coefficients in Equation (32), the MI spectral density is approximately equal to the normalized SINR spectral density via Taylor series approximation. However, for frequencies with large coefficients, the MI spectral density is approximately the logarithm of the normalized SINR spectral density. The logarithm function lowers the values of these large coefficients. This effect allows for less dominant frequency components in the MI spectral density to be somewhat significant.

**Figure 4:**
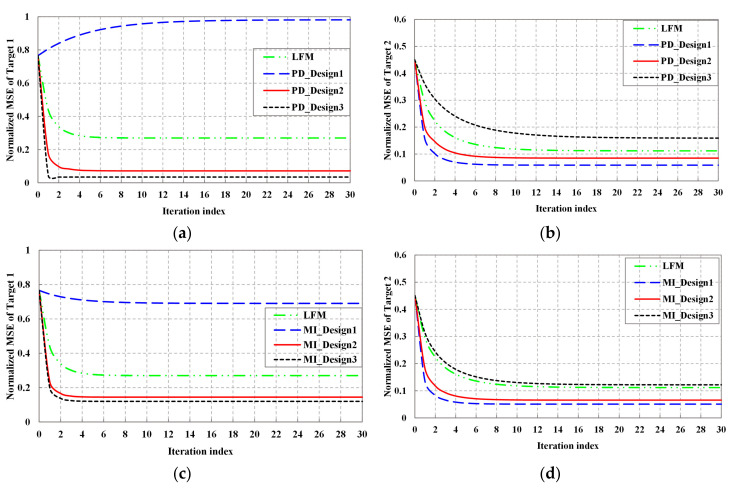
Normalized MSE performance: (**a**) Normalized MSE performance of detection probability-based waveform of Target1; (**b**) Normalized MSE performance of detection probability-based waveform of Target2; (**c**) Normalized MSE performance of MI-based waveform of Target1; (**d**) Normalized MSE performance of MI-based waveform of Target2.

Figure 4 depicts the relation between normalized MSE of estimated TSC and KF iteration for Target1 and Target2, respectively, where the MSE is defined in Equation (9). Since the initial Bayesian estimation is not quite accurate, the initial MSE of both the LFM and designed waveforms are relatively high. In Figure 4a,c, for Target1, with the increasing of tracking number, the normalized MSE of LFM, Design2, and Design3 waveforms decreases, then tends to flat because of the accumulation of received data. By using KF, each round of iteration brings the estimated TIR into better agreement with the actual TIR. Thus, Kalman filter can significantly improve the estimation accuracy. It can be obtained that the normalized MSE of Design3 waveform is far less than that of fixed LFM waveform. Moreover, the iteration number of Design3 waveform is fewer than that of LFM waveform when the normalized MSE tends to flat. That is to say, CR with adaptive waveform can track target quicker than traditional radar with fixed waveform. However, it is interesting to note that compared with LFM waveform, the normalized MSE of Design1 waveform increases. Since the weight vector is $\alpha ={\left[0,1\right]}^{T}$, the power spectrum distribution of Design1 waveform tends to concentrate in bands where the power of Target2 is large. Thus, for Design1 waveform, the normalized MSE of Target1 is large, nevertheless, the normalized MSE of Target2 is small. It is well validated by Figure 4b,d. In addition, in Figure 4a,c, it can be obtained that the normalized MSE of MI_Design1 waveform is less than that of PD_Design1 waveform, since the energy distribution of MI_Design1 waveform is evenly than that of PD_Design1 waveform.

**Figure 5:**
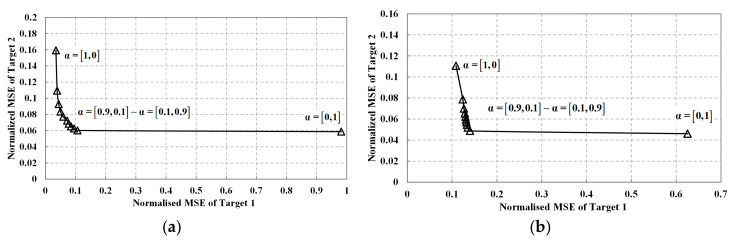
Joint normalized MSE performance: (**a**) Joint normalized MSE performance of detection probability-based waveform; (**b**) Joint normalized MSE performance of MI-based waveform.

Figure 5 depicts the joint normalized MSE performance of Target1 and Target2. For the joint waveform design consideration, the weight vector α is adopted to control the importance of different targets. By turning the weight coefficients, we can obtain different MSE performance for different targets. In Figure 5a,b, it is obvious that for both detection probability-based waveform and MI-based waveform, the points at the lower-left corner have the best joint normalized MSE performance. Thus, the joint MSE can be effectively reduced by the proposed method.

**Figure 6:**
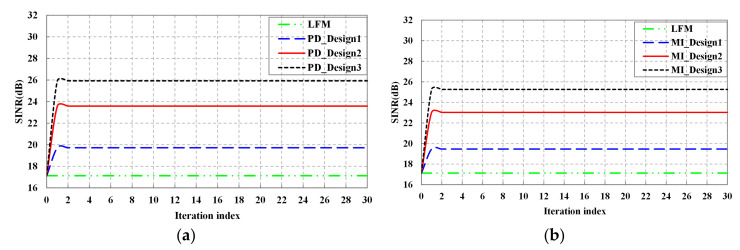
SINR performance: (**a**) SINR performance of detection probability-based waveform; (**b**) SINR performance of MI-based waveform.

Figure 6 shows the SINR performance of the detection probability-based optimal waveform and the MI-based optimal waveform, respectively. The performance of LFM waveform is utilized for comparison. As expected, with the increase of tracking number, the SINR of both detection probability-based optimal waveform and the MI-based optimal waveform increases, then tends to be flat. Moreover, it can be obtained that the SINR performance of Design3 waveform is better than that of Design1 waveform. As it is known from Figure 3, the power of Target1 is stronger than that of Target2, thus, the echo of Design3 waveform contains more target information. In addition, the SINR of detection probability-based optimal waveform is slightly higher than that of MI-based optimal waveform under the same transmit energy constraint. But the SINR of both of detection probability-based optimal waveform and the MI-based optimal waveform are higher than LFM waveform, especially when the tracking number is high.

**Figure 7:**
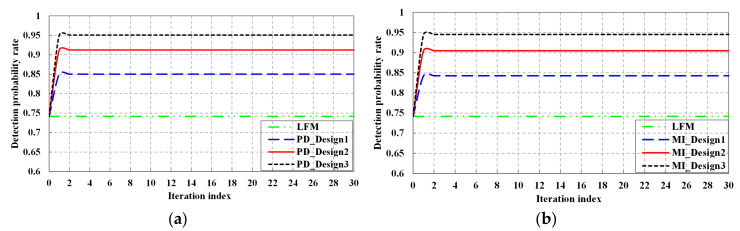
Detection performance: (**a**) Detection performance of detection probability-based waveform; (**b**) Detection performance of MI-based waveform.

In Figure 7, we investigate the detection probability rate performance of the detection probability-based optimal waveform, the MI-based optimal waveform, and LFM waveform. The false alarm probability is $P_{FA}=10^{-6}$. We may obtain that except for the LFM waveform, the detection probability performance of both detection probability-based optimal waveform and the MI-based optimal waveform are improved with the increase of KF iterations. From Equation (23), since the detection probability is a non-decreasing function of the SINR under the log-likelihood ratio test, the trend of detection probability is consistent with that of SINR.

**Figure 8:**
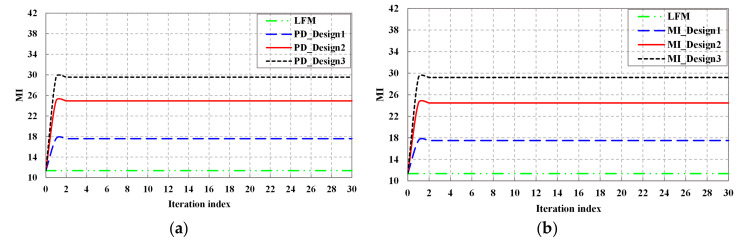
MI performance: (**a**) MI performance of detection probability-based waveform; (**b**) MI performance of MI-based waveform.

Figure 8 shows the MI performance of the detection probability-based optimal waveform, the MI-based optimal waveform, and LFM waveform. It can be observed that the MI performance of both detection probability-based optimal waveform and the MI-based optimal waveform are improved with the increase of KF iteration, except for LFM waveform. From Equation (32), since the MI spectral density is a non-decreasing function of the normalized SINR spectral density, the trend of MI is consistent with that of SINR.

## 5. Conclusions

The application of CR to waveform design is creating advances in target estimation and detection through the adaptive handling of uncertainties in the target and the environment. In this paper, based on multiple extended targets with unknown TIR, an improved waveform optimization design method for target detection is proposed in signal-dependent interference, as well as additive channel noise. A weight vector is adopted to control the importance of different targets. The whole signal processing model is investigated in Fourier domain. Based on extended targets with unknown TIR, Kalman filter is used to track the extended targets for estimating the TIR. Ensuring the TIR estimation precision, the transmit waveform is optimally determined at each step based on the previous step. In particular, under the same constraint on waveform energy and bandwidth, the MI method is also considered. The relationship between the waveforms that are designed based on the two criteria is discussed.

Simulation results demonstrate that Kalman filter can significantly improve the estimation accuracy. The spectrum distribution of both detection probability-based waveform and MI-based waveform is mainly concentrated in bands where the target power is large and the clutter power is small. This will greatly reduce the influence of interference on echoes. Therefore, the SINR, detection probability, and MI performance can be improved significantly. Among them, the SINR performance is improved at least 17.6% by the proposed method with detection probability-based waveform and MI-based waveform compared with that of LFM. The detection probability performance is improved at least 13.3% by the proposed method with detection probability-based waveform compared with the one with LFM. The MI performance of MI-based waveform is at least 50% better than that of LFM. Moreover, it is interesting to note that the detection probability-based waveform tends to concentrate the main energy in a few narrow frequency bands. The MI-based waveform tends to distribute its energy over some relatively wide bands. Besides, the joint MSE can be effectively reduced by the proposed method. Above all, CR with proposed waveforms outperforms traditional radar with fixed LFM waveforms significantly in estimation and detection performance. Moreover, since both the estimate of TIR and transmitted waveform of CR can be updated according to the environment information fed back by receiver, the proposed waveform optimization design method is more practical and flexible than traditional radar waveform design method. During the future work, more constrains, such as compromise considering target detection and recognition, need to be taken into account for the waveform optimization. In addition, the whole waveform optimization design approach will be investigated in real conditions in the future.

## Figures and Tables

**Table:** 

Waveform	$\alpha_{1}$	$\alpha_{2}$
Design1	0	1
Design2	0.5	0.5
Design3	1	0

**Table 1:** The parameter of designed waveforms.

**Waveform**	$\alpha_{1}$	$\alpha_{2}$
Design1	0	1
Design2	0.5	0.5
Design3	1	0
